# F *K*-edge XAS as a tool to examine F environments in complex inorganic systems

**DOI:** 10.1107/S1600577526002821

**Published:** 2026-04-10

**Authors:** Malin C. Dixon Wilkins, John M. Bussey, John S. McCloy

**Affiliations:** a School of Mechanical and Materials Engineering Washington State University Pullman WA USA; Advanced Photon Source, USA

**Keywords:** soft X-ray spectroscopy, XAS, inorganic materials

## Abstract

As part of an effort to establish the principles and application of F *K*-edge X-ray absorption spectra to complex inorganic systems, the F *K*-edge spectra of a range of glasses, minerals and synthetic crystalline compounds are presented and discussed in terms of the F environments present. Crystalline fluorides often display richly detailed spectra as a result of the small core-hole broadening at the F *K*-edge, and the analysis of the spectra is presented by way of examining trends in material structure and bonding, spectral fingerprinting, and linear combination fitting. The benefits and drawbacks of each method of analysis are discussed, and recommendations for collection of high quality spectra are presented. The speciation of F in three oxide glasses is established by comparison of the observed F *K*-edge spectra with the spectra of suitable crystalline reference material. The F environments determined this way are in excellent agreement with previous works that utilized other methods of probing the F speciation, though in one case ambiguity in the observed spectrum does prevent definitive identification. F *K*-edge spectra were also calculated using the *FEFF* software package, with the aim of calculating spectra that reproduce the experimental spectra accurately with a minimum of prior knowledge and assumptions. The spectrum of CaF_2_ was calculated with a wide range of simulation parameters, and comparisons with experimental data are made to facilitate a simple computational workflow, that is validated by application to the simulation of the spectrum of Ca_4_Si_2_O_7_F_2_. Observations and recommendations are made that will assist other experimenters in the collection and analysis of future high-quality F *K*-edge spectra. The data and analyses presented establish F *K*-edge X-ray absorption spectroscopy as a powerful tool for the characterization of F speciation and environments in complex inorganic materials.

## Abstract 2

Experimental and computational F *K*-edge X-ray absorption spectra of a range of complex inorganic materials are presented and analysed, with discussion as to what information on F speciation and environment can be obtained from the spectra. Observations and recommendations are made that will assist future research in the collection and analysis of high-quality F *K*-edge spectra.

## 1. Introduction

The chemistry of inorganic fluoride-containing materials has key importance in a range of industries. For example, the Hall–Héroult process is the major industrial process for production of metallic Al; it involves dissolution of Al_2_O_3_ (refined from Al ores such as bauxite) into molten mixtures of NaF/AlF_3_ (the bulk of which is from cryolite, Na_3_AlF_6_), followed by electrolysis in the presence of solid C, resulting in metallic Al (Lumley, 2010 ▸). In recent years, there has been significant renewed interest in molten salt reactors for civil nuclear power generation, where the molten salts act as the primary coolant. In some designs the fuel is also dissolved in the salt, permitting on-line refuelling operations. For both applications, fluoride salt systems are among the more promising candidates, including 66LiF·34BeF_2_ (FLiBe), 46.5LiF·11.5NaF·42KF (FLiNaK), 61LiF·39NaF (FLiNa), and NaF–ZrF_4_ mixtures (Roper *et al.*, 2022 ▸; Gandy, 2019 ▸). Inorganic F-containing materials are also important for dental materials, including simple binary fluorides such as SnF_2_ or NaF, as well as more complex systems such as the fluoride-containing silicate glass component of glass-ionomer dental cements (Wiegand *et al.*, 2007 ▸; Burke *et al.*, 2006 ▸). Fluorinated glasses, generally phosphate–silicate, have also been investigated for their bioactivity (Gharbi *et al.*, 2023 ▸; Christie *et al.*, 2011 ▸; Shah, 2016 ▸). A wide variety of inorganic fluoride and oxyfluoride glasses and glass ceramics are also important functional materials, including optical materials (Poulain, 2024 ▸; Galdo *et al.*, 2025 ▸; Annunziato *et al.*, 2022 ▸), laser media (Reben & Środa, 2013 ▸; Bansal *et al.*, 2024 ▸), scintillators (Guo *et al.*, 2024 ▸; Sun *et al.*, 2023 ▸; Liu *et al.*, 2023 ▸), ion conductors (Ali Nowroozi *et al.*, 2021 ▸; Patro & Hariharan, 2013 ▸), and many more. For these examples and other applications, a solid understanding of the chemistry and behaviour of inorganic fluorides is important.

A key method for examining F local environments is ^19^F nuclear magnetic resonance (NMR) spectroscopy. Solid state magic angle spinning (MAS) NMR and related techniques have been extensively utilized to examine F chemistry, including for inorganic systems where F plays a significant structural role, for example in silicate, phosphate and fluoride glasses (Youngman, 2018 ▸; Stebbins & Zeng, 2000 ▸; Hill *et al.*, 2006 ▸; Stamboulis *et al.*, 2005 ▸; Schaller *et al.*, 1992 ▸; Brow *et al.*, 1992 ▸; Chen *et al.*, 2022 ▸), minerals (Zhou *et al.*, 2007 ▸; Tossell & Liu, 2004 ▸; Huve *et al.*, 1992 ▸; Labouriau *et al.*, 1995 ▸), and synthetic fluorides (Bessada & Anghel, 2003 ▸; Kiczenski & Stebbins, 2002 ▸; Sadoc *et al.*, 2011 ▸; Martel *et al.*, 2018 ▸). However, significant drawbacks do exist, including long data collection times, large sample sizes, the high level of required operator expertise, and interference from paramagnetic nuclei. The use of F *K*-edge X-ray absorption spectroscopy (XAS) as an alternative method to examine F environments in complex inorganic materials is not yet fully established. Compared with solid state ^19^F NMR, data collection times are significantly shorter, instrument operation is simpler, and much less material is needed (samples less than 50 mg are easily examined). A further advantage of X-ray spectroscopic techniques is the use of microfocus techniques, where a micrometre-sized beam can be utilized to gain spatially resolved information in heterogeneous systems in tandem with X-ray fluorescence (XRF) mapping. The need for very bright tuneable X-ray sources should be noted as a significant drawback for routine analysis, as soft XAS studies (including probing the F *K*-edge) usually require the use of synchrotron lightsources.

The use of F *K*-edge spectroscopy has been reported for a variety of systems, albeit with few total spectra compared with other elements. Microfocus XRF mapping has been utilized to examine the presence of per- and poly-fluoro­alkyl substances (PFAS) in a variety of environmental and consumer products, with corresponding F *K*-edge XANES utilized to determine the species present (Roesch *et al.*, 2023 ▸). Work examining the presence of PFAS in soils and sewage sludges (Roesch *et al.*, 2022 ▸), surfactant modified clay (Yan *et al.*, 2021 ▸), and wastewater-derived fertilisers (Vogel *et al.*, 2023 ▸) has also been performed, with a focus on determining the speciation and distribution of F. A significant body of theoretical and experimental work exists examining binary fluorides, with a particular focus on the alkali and alkaline-earth fluorides (Oizumi *et al.*, 1985 ▸; Nakai *et al.*, 1986 ▸), though a number of transition metal (Vinogradov *et al.*, 2005 ▸; Sanz-Matias *et al.*, 2022 ▸) and other metal fluorides (Schroeder & Weiher, 2006 ▸; Ward *et al.*, 2017 ▸) have been reported.

Despite the amount of research utilizing F *K*-edge XAS, there is a lack of investigation of complex inorganic fluoride systems, for example silicate and fluoride glasses, minerals, and synthetic mixed fluorides. In this work we report the spectra of a range of binary and ternary fluoride compounds, and fluorine-containing glasses and minerals, and discuss the methods of XAS data collection and analysis as applied to the F *K*-edge of these and similar systems.

## 2. Methodology

F *K*-edge spectra were collected at the Spherical Grating Monochromator (SGM) beamline at the Canadian Light Source (CLS), Saskatoon, SK, Canada. Materials were prepared as powders (<20 mg) spread on conductive carbon tape, which were then mounted on Al plates held at 45° to the incident beam. Spectra were collected between 680 eV and 730 eV, with both fluorescence (partial fluorescence yield, PFY) and drain current (total electron yield, TEY) measurements made simultaneously. For each sample, 10 to 20 scans of 60 s each were performed, with the sample position moved after each scan to reduce the possibility of beam damage, for total data collection times of 15–30 min per sample. Spectra were normalized compared with the incident flux and calibrated on the absolute energy scale by comparison of the spectrum of CaF_2_ with that of Oizumi *et al.* (1985 ▸), who reported a transmission mode spectrum for CaF_2_; a shift of −1.1 eV from this reference was observed and used as a correction for the spectra reported here. CaF_2_ spectra were collected periodically throughout the experiment to ensure no drift in beam energy was observed. Data reduction, analysis and linear combination fitting were performed in the *Larch* software suite (Newville, 2013 ▸).

Samples of high purity binary fluorides LiF, NaF, KF, RbF, CsF, MgF_2_, CaF_2_, SrF_2_, BaF_2_, SnF_2_, two samples of AlF_3_ (one determined to be α-AlF_3_ and the other β-AlF_3_·3H_2_O), Na_2_PO_3_F, and KBF_4_ were obtained as reagent grade chemicals, see the supporting information. Several more complex fluorides were synthesized: β-CaAlF_5_, Ca_2_AlF_7_ (synthetic sbacchiite), Ca_4_Si_2_O_7_F_2_ (synthetic cuspidine), and FLiNa (the eutectic salt composition at 61 mol.% LiF, 39 mol.% NaF); the synthetic details and crystallographic identities as-checked by X-ray diffraction (XRD) are provided in the supporting information. Samples of the natural fluorides cryolite (Na_3_AlF_6_) and chiolite (Na_5_Al_3_F_14_), both from Greenland, were also examined. Detailed characterization of these minerals was provided in a previous ^19^F study (McCloy *et al.*, 2024 ▸).

The use of F *K*-edge XAS to examine the environment of F in glasses is another application. In this work, three glasses were prepared using compositions from previous works where characterization of the F environment was reported by other methods. Since glassy materials lack long-range order, it was anticipated that the F *K*-edge spectra of F in different amorphous systems would display variation in the position and shape of the white line, though with few features observed at higher energies. The three glasses considered here are (with nominal, as batched compositions in parenthesis): (1) SSPN15 (mol%, 10SnO–40SnF_2_–35P_2_O_5_–15NaF) (Chen *et al.*, 2022 ▸); (2) Ca-silicate glass (mol%, 39.2CaO–58.9SiO_2_–2.0CaF_2_, here called CS-Stebbins) (Stebbins & Zeng, 2000 ▸); (3) an Li–Pb–B oxyfluoride glass (mol.% 50B_2_O_3_–10PbO–40LiF, here called LPB40) (Cattaneo *et al.*, 2008 ▸). Synthesis details for these glasses can be found in the supporting information.

Simulations of F *K*-edge spectra were performed using the *FEFF10* (Kas *et al.*, 2021 ▸) software package. Several CaF_2_ spectra were calculated using different parameters to evaluate the applicability of variables within the FEFF XANES calculation workflows; full details are given below, and example *FEFF* input files are given in the supporting information.

## 3. Results

### 3.1. Experimental spectra

#### 3.1.1. Alkali and alkaline-earth binary fluorides

During data collection for the alkali and alkaline-earth binary fluorides, it was noted that the PFY data were significantly affected by over-absorption (also called self-absorption), leading to significant suppression of the most intense features (Tröger *et al.*, 1992 ▸). This was also observed for many of the other high F-content materials studied and usually led to only the TEY signal being a fair representation of the actual absorption of the material (see Fig. S1 of the supporting information).

**Figure 1:**
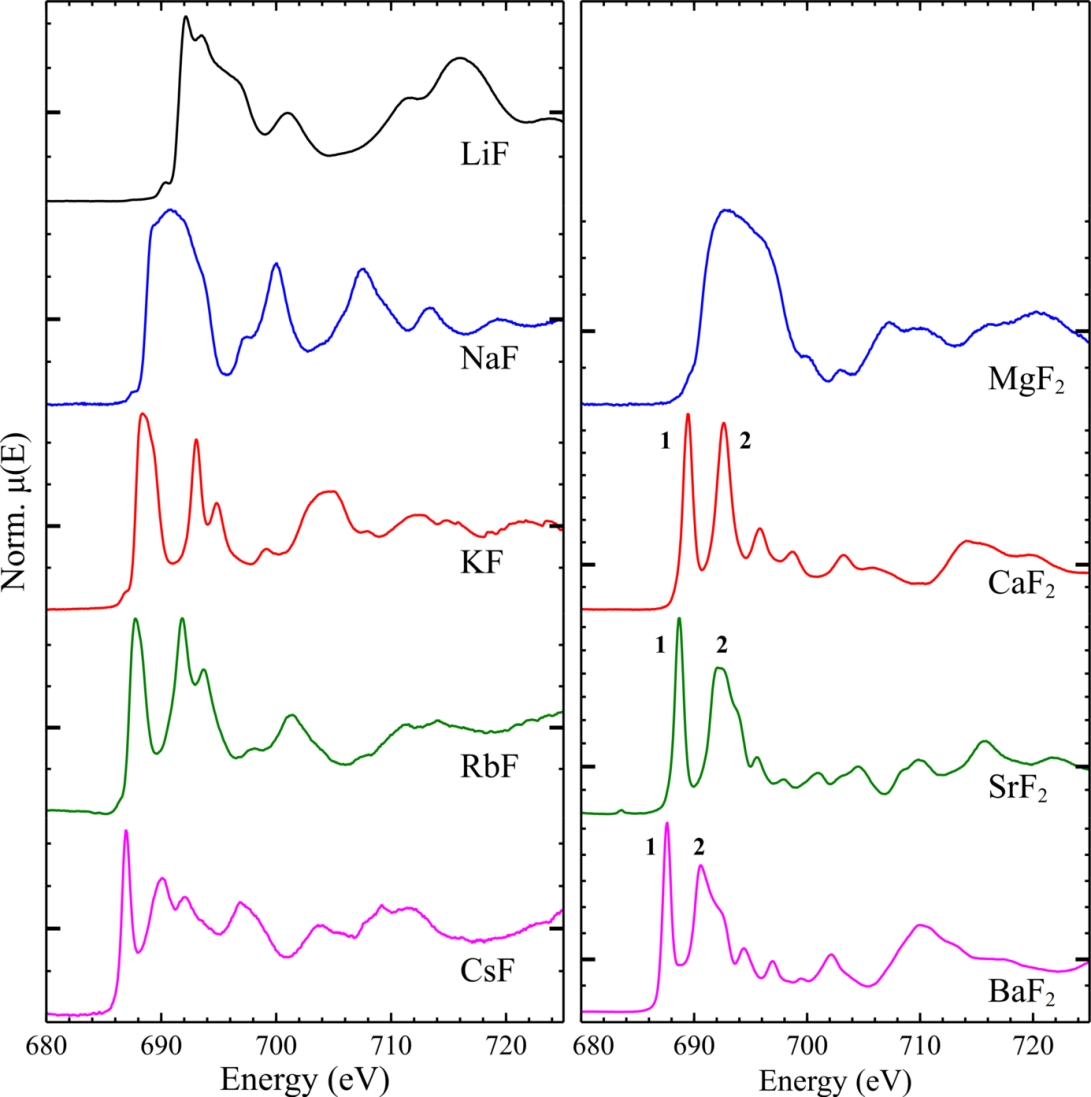
Normalized TEY F *K*-edge spectra of the alkali (left) and alkaline-earth (right) binary fluorides. Each spectrum is presented on a unique *y*-axis, with μ(*E*) = 1 marked with a larger tick.

The spectra observed for the alkali (LiF, NaF, KF, RbF, CsF) and alkaline-earth (MgF_2_, CaF_2_, SrF_2_, BaF_2_) binary fluorides were in good agreement with those previously reported (Oizumi *et al.*, 1985 ▸; Nakai *et al.*, 1986 ▸; Sanz-Matias *et al.*, 2022 ▸), see Fig. 1 ▸. In both series, a systematic shift in edge position was observed, with larger, higher atomic number (*Z*) cations resulting in lower energy edge positions. This can be ascribed to increasing ionicity of the *M*—F bonding leading to a higher effective charge on the F, which in turn results in a decrease in the binding energy of the 1*s* electrons (Oizumi *et al.*, 1985 ▸; Nakai *et al.*, 1986 ▸).

For the alkaline-earth fluorides, the observed spectral shapes follow what might be expected from the crystal structures of the materials: MgF_2_ is rutile structured, whilst CaF_2_, SrF_2_ and BaF_2_ are fluorite structured. This has previously been discussed in terms of crystal structure differences only (Oizumi *et al.*, 1985 ▸), though a more comprehensive explanation must also account for differences in F 2*p* and metal 3*d* orbital hybridization, with MgF_2_ (and NaF, LiF) having no hybridization as a matter of course (Sanz-Matias *et al.*, 2022 ▸). The spectra of the fluorite structured materials displayed consistent features, though with some small relative shifts, with a major shared characteristic of two intense features: **1**, at the white line, and **2**, approximately 3.2 eV above the white line. In the spectra of SrF_2_ and BaF_2_, a shoulder was present on the high energy side of **2** that was not observed for the isostructural CaF_2_.

The spectra observed for the alkali fluorides show significant differences in spectral shape between LiF, NaF, and the heavier alkali elements. This variation was observed despite the series of alkali fluorides all forming in the rock salt structure; this suggests that the differences in spectral shape must be due to contributions from electronic effects rather than structural effects only. This is well explained by the trends in F 2*p* and metal 3*d* hybridization mentioned above and posited previously (Sanz-Matias *et al.*, 2022 ▸).

Fingerprinting, *i.e.* visual comparison of the XANES region with materials of known oxidation state and coordination environment, constitutes an important method of analysis for many XAS studies. As core-hole broadening magnitude is generally correlated with the atomic number of the absorber (Keski-Rahkonen & Krause, 1974 ▸), spectral fingerprinting is especially useful for the less broadened edges belonging to low-*Z* elements. It should be obvious on examination of the richly featured spectra seen here that fingerprinting may be usefully applied to the F *K*-edge of these and related systems.

#### 3.1.2. Mixed cation fluorides

**Figure 2:**
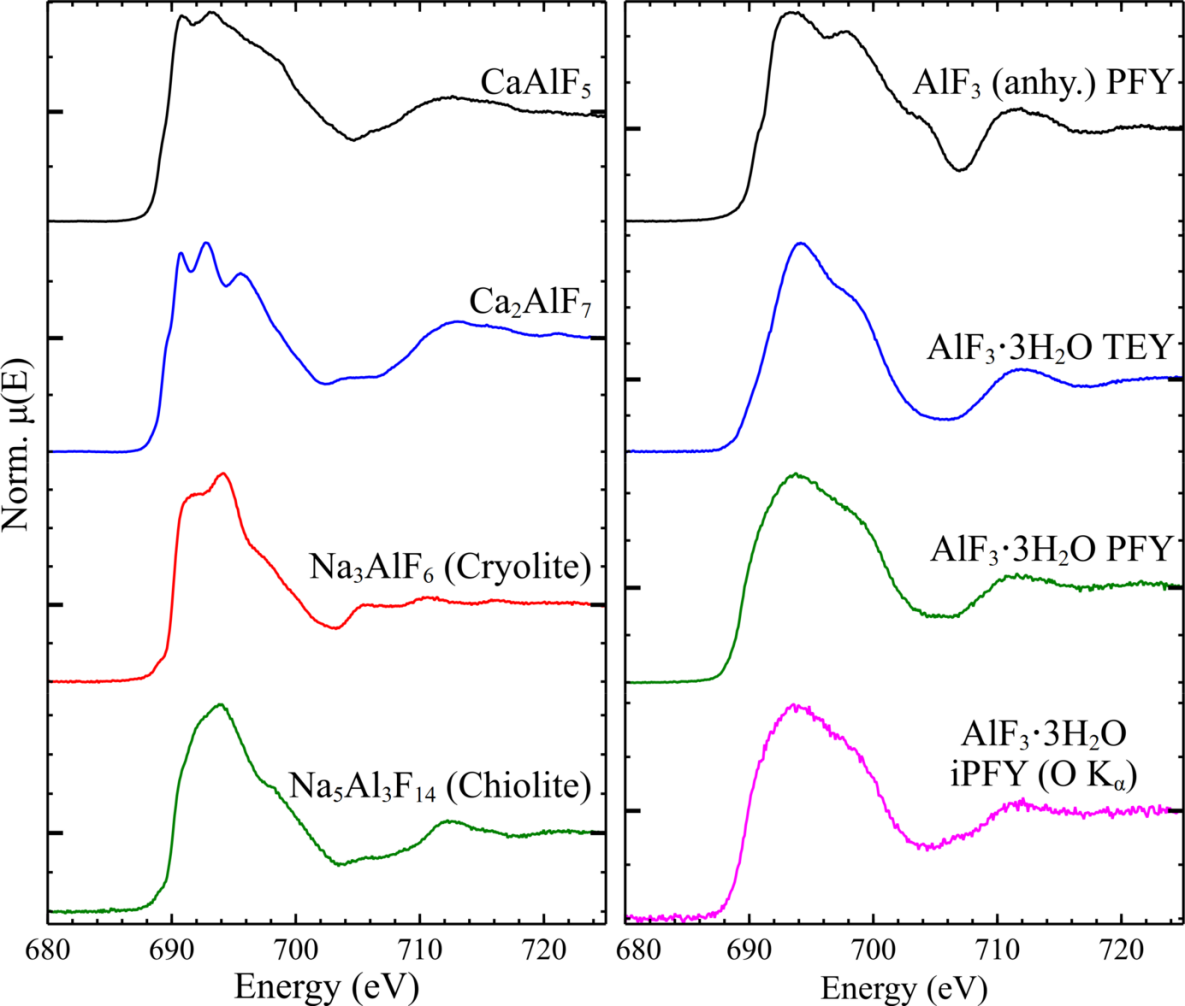
(Left) Normalized TEY F *K*-edge spectra of synthetic β-CaAlF_5_ and Ca_2_AlF_7_, and mineralogical samples of cryolite (Na_3_AlF_6_) and chiolite (Na_5_Al_3_F_14_). (Right) F *K*-edge spectra of AlF_3_ (PFY) and AlF_3_·3H_2_O (TEY, PFY and O *K*-edge iPFY). Each spectrum is presented on a unique *y*-axis, with μ(*E*) = 1 marked with a larger tick.

In order to examine the impact of similar but varied structures with F coordination environments more complex than those of the alkali and alkaline-earth fluorides, the F *K*-edge spectra of β-CaAlF_5_, Ca_2_AlF_7_, cryolite (Na_3_AlF_6_) and chiolite (Na_5_Al_3_F_14_) were collected, see Fig. 2 ▸.

β-CaAlF_5_ has five unique F sites, all of the same multiplicity (*i.e.* number per unit cell) (Body *et al.*, 2005 ▸): two sites trigonal planar 1Al, 2Ca, one is trigonal planar 2Al, 1Ca, and two are linear 1Al, 1Ca. Ca_2_AlF_7_ also has five unique F sites (Domesle & Hoppe, 1980 ▸), two of multiplicity eight (both trigonal planar, 1Al, 2Ca), three of multiplicity four (trigonal planar 1Al, 2Ca, near-linear 1Al, 1Ca, and trigonal planar 3Ca). The spectra of the two are clearly similar, with overall spectral envelopes of very similar shape, though Ca_2_AlF_7_ has three distinct features on the white line, where CaAlF_5_ only has two less distinct features. Compared with the spectra of the binary fluorides discussed above, the spectra are less clearly featured, with a broad white-line dominating due to configurational averaging over the five unique F environments in each. The predominance of a trigonal planar F–1Al, 2Ca environment in Ca_2_AlF_7_ (20 of the 28 F per unit cell) is likely the reason for the more distinct white-line features compared with those of CaAlF_5_, where a wider distribution of F environments is present, particularly given the presence of a small fraction of Ca_2_AlF_7_ in the phase assemblage of this material (see supporting information).

The spectra of cryolite (Na_3_AlF_6_) and chiolite (Na_5_Al_3_F_14_) also have broadly similar overall spectral envelopes with a notable difference in the shape of the white-line: both have maxima at ∼694.0 eV, but the rising edge of cryolite is steeper, resulting in a more prominent shoulder at ∼691.2 eV on the low-energy side. The major F environments in both are (distorted) tetrahedra with coordination by 3Na, 1Al (8 of 12 F in cryolite, 16 of 28 in chiolite), with distorted square pyramidal coordination by 4Na, 1Al also present in both (4 of 12 F in cryolite, 4 of 28 in chiolite) (Yang *et al.*, 1993 ▸; Jacoboni *et al.*, 1981 ▸). Chiolite has an additional near-linear bridging Al—F—Al coordination (8 of 28 F), which directly relates to the difference in AlF_6_ connectivity in the two structures: in cryolite the AlF_6_ octahedra are not directly linked, with connection to Na polyhedra only; in chiolite AlF_6_ octahedra are corner sharing, forming an open sheet linked by layers of the Na polyhedra (Hawthorne & Herwig, 2021 ▸). As above, it is possible that the slightly less distinct features in the spectrum of chiolite compared with cryolite is due to more variation in the F environments present.

There are many polymorphs of both anhydrous and hydrous AlF_3_, but all of them are structurally based on AlF_6_ octahedra with bridging F atoms between octahedra (Kemnitz *et al.*, 2006 ▸; Daniel *et al.*, 1990 ▸). Included among the polymorphs are those which occur as the natural minerals oskarssonite (α-AlF_3_) and rosenbergite (β-AlF_3_·3H_2_O) (Jacobsen *et al.*, 2014 ▸; Olmi *et al.*, 1993 ▸). Initial examination of the PFY spectrum of β-AlF_3_·3H_2_O (see Fig. 2 ▸) suggested that some amount of over-absorption had occurred, with a broader white-line of lower intensity compared with the TEY spectrum of the same material, though the same features were apparent in both. It was hoped that, by using the inverse partial fluorescence yield (iPFY) method of detection (Achkar *et al.*, 2011 ▸), the F *K*-edge spectrum could be indirectly measured by examining the change in intensity of the O *K*α emission without the impact of over-absorption seen in the standard PFY detected spectrum. iPFY measurements monitor the intensity of a (non-resonant) fluorescent emission (in this case, O *K*α at 524.9 eV) at an energy lower than the emission of the absorbing (in this case, F *K*α at 676.8 eV) as the incident energy is scanned across the energy range of interest (see Fig. S2). The intensity of the non-resonant emission varies as a function of the overall attenuation length into the sample, and so is inversely proportional to the absorption coefficient, μ(*E*), at each energy. However, it was apparent that the O *K*α iPFY spectrum much more closely resembled the standard PFY spectrum and still appeared broadened (or the features were otherwise suppressed) relative to the TEY spectrum. Given that iPFY spectra should not be impacted by over-absorption (Achkar *et al.*, 2011 ▸), the cause of this is unclear and will be discussed further below.

#### 3.1.3. FLiNa and linear combination fitting

As the authors have reported previously (McCloy *et al.*, 2025 ▸), the F *K*-edge spectrum of FLiNa (the eutectic composition at 61 mol.% LiF, 39 mol.% NaF) strongly resembles a combination of the spectra of the components LiF and NaF. The diffraction pattern of solid FLiNa showed the presence of LiF and NaF only, in agreement with previously reported phase equilibria (Sangster & Pelton, 1987 ▸).

**Figure 3:**
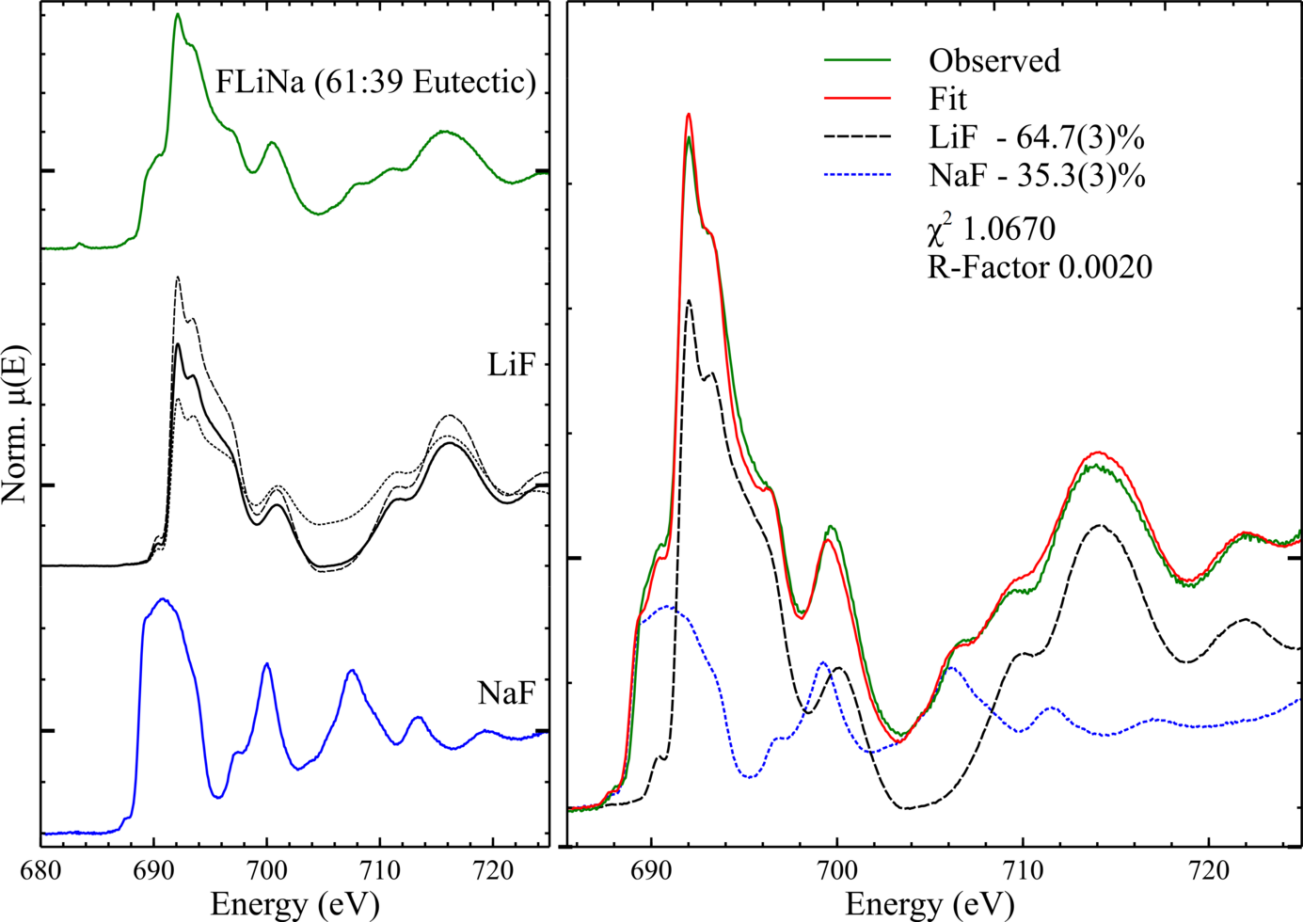
(Left) Normalized TEY spectra of FLiNa (61:39 molar ratio of LiF and NaF, respectively), LiF (with three different choices of normalization) and NaF. (Right) LCF of the spectrum of FLiNa, with as-fit spectral fractions and goodness-of-fit parameters listed. Each spectrum is presented on a unique *y*-axis, with μ(*E*) = 1 marked with a larger tick.

Another common method of XANES analysis is linear combination fitting (LCF), where the spectrum under examination is fit by a linear combination of two or more reference spectra. The normalized FLiNa spectrum was fit by linear combinations of the spectra of LiF and NaF (see Fig. 3 ▸), under the constraint that the sum of the component spectral weights equalled 100%; no energy shift of the spectra was permitted. The best fit comprised 64.7 (3)% LiF and 35.3 (3)% NaF, in reasonable agreement with the expected stoichiometry, though it should be noted that the quoted uncertainty is a statistical uncertainty only, and does not represent the total uncertainty in these values, which is likely to be significantly higher due to other, more difficult to determine, contributions.

During LCF it was noted that the exact weights of the component spectra in the final fit varied significantly as a function of the data normalization parameters used, massively increasing the analysis uncertainty for this example. LiF, in particular, displays a complex spectral shape, with large, unevenly spaced maxima and minima; for comparison, see, for example, NaF, where the post-white-line oscillations are approximately evenly spaced. This complex spectral envelope makes the choice of normalization difficult, with three examples of normalized spectra of LiF shown in Fig. 3 ▸; all three spectra seem to be reasonable normalized representations of the raw data when examined alone, but there is no strict reason for any one to be chosen over the others. This ambiguity in choosing a consistent normalization procedure, across what can be significantly varied pre-edge and post-edge regions, is compounded by the more complex spectral shapes seen for the alkali and alkaline-earth binary fluorides, though this is less of an issue for spectra with simpler post-edge shapes (*e.g.* cryolite, Na_3_AlF_6_ in Fig. 2 ▸).

Given the complex spectral shapes shown here, it is apparent that LCF, and related methods such as principal component analysis (PCA) and iterative target transformation factor analysis (ITFA), form useful data analytical techniques that can be applied to the F *K*-edge. However, as shown above, care must be taken during data reduction and normalization, even more so than typical higher energy edge XAS measurements, where these same complex spectral shapes can end up causing significant uncertainty that may not otherwise be apparent from fitting statistics.

#### 3.1.4. F in amorphous systems

**Figure 4:**
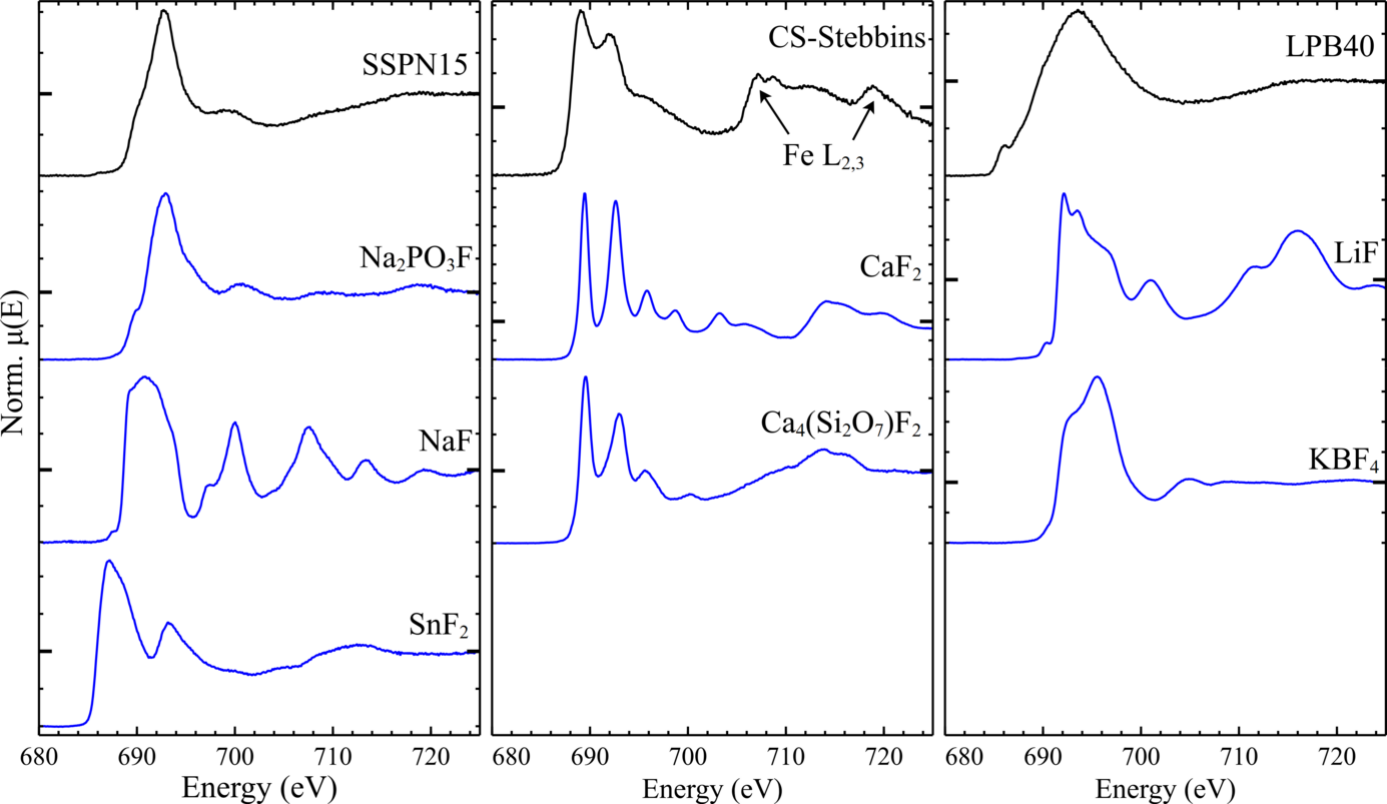
Normalized F *K*-edge spectra of three glasses, SSPN15 (left), CS-Stebbins (centre), LPB40 (right) (see text for compositions), alongside a range of synthetic crystalline reference compounds. The spectra of the glasses, Na_2_PO_3_F, SnF_2_ and Ca_4_Si_2_O_7_F_2_, are PFY-detected; the spectra of NaF, CaF_2_, LiF and KBF_4_ are TEY-detected. Each spectrum is presented on a unique *y*-axis, with μ(*E*) = 1 marked with a larger tick.

The F *K*-edge spectra of the three glasses are shown in Fig. 4 ▸, alongside a number of synthetic crystalline reference compounds. The spectrum of SSPN15 (Sn–Na–P–O–F) was notably similar to that of compound Na_2_PO_3_F (short F—P bond, with one or two near-neighbour Na) with a single white-line feature at ∼692.8 eV followed by a second lower intensity feature at ∼699.5 eV (SSPN15) or 700.4 eV (Na_2_PO_3_F). In comparison, the spectra of NaF (F–6Na) and SnF_2_ (F–3Sn, varied F—Sn distances for the four unique F sites) display white lines of significantly differing shape and position. It is apparent based on these spectra that the dominant F environment in SSPN15 is close to that in Na_2_PO_3_F, where F forms part of PO_3_F tetrahedra; in Na_2_PO_3_F these tetrahedra are isolated (Durand *et al.*, 1974 ▸), but in SSPN15 form the partially polymerized glass network. The asymmetry seen on the low energy side of the white line of SSPN15 is more distinct than that of Na_2_PO_3_F, which may relate to the presence of a small fraction of F in coordination environments more like those in NaF or SnF_2_. The white line of SSPN15 does not appear significantly broadened compared with that of Na_2_PO_3_F, perhaps suggesting that F is present in only a limited range of different environments. This is in excellent agreement with the F–1*s*X-ray photoelectron spectrum of SSPN15 reported by Chen *et al.* (2022 ▸), with the spectrum dominated by the feature assigned to F—P species, though a smaller contribution from F—Sn species is also apparent.

The spectrum of a simple Ca–silicate glass (Ca–Si–O–F) comprised a white line of two distinct features at 689.0 eV and 692.0 eV, with a lower intensity feature/shoulder seen at 696.3 eV. The Fe *L*_3_ and *L*_2_ absorption edges (maxima at 707.2 eV and 718.9 eV, respectively) were evident, since Fe was present as a contaminant from the SiO_2_ precursor. This motif of two white-line features followed by a lower intensity feature is similar to the first three features observed for both CaF_2_ and synthetic cuspidine (Ca_4_Si_2_O_7_F_2_, two distinct F sites), both of which contain F coordinated tetrahedrally by Ca. The impact of the disordered nature of the glass is obvious, especially compared with SSPN15, see above, with the observed broadening caused by the presence of a range of F—Ca angles and distances, and likely also some variation in F—Ca coordination number. These observations are consistent with those made by Stebbins & Zeng (2000 ▸) using ^19^F NMR, who observed a single, though significantly broadened by structural disorder, F environment shifted slightly from that of F in crystalline CaF_2_.

The final glass, a Li–Pb–B oxyfluoride (LPB40), showed an F *K*-edge spectrum which was significantly broadened, even compared with the other glasses presented here (see Fig. 4 ▸), with only two features apparent: the broad white-line and a low intensity shoulder on the rising edge. Unlike SSPN15 and CS-Stebbins, there is no clear relation between the spectrum of the glass and the crystalline references LiF (F–6Li) and KBF_4_ (short F—B with 3 K further away, significantly varied F—K distances). From inspection of the spectra, it is feasible that the F environment in LPB40 is a mix of those seen in LiF and KBF_4_, but the lack of features of the LPB40 F *K*-edge spectrum means no unambiguous determination can be made, especially without the spectrum of a lead fluoride for comparison. In the work by Cattaneo *et al.* (2008 ▸), ^19^F MAS-NMR showed two distinct F environments, assigned to an LiF-like environment (∼60% of the F), and F bonded to B (∼40%).

It should be noted that some fluorine volatilization is expected from the glass melts above, and the compositions are taken as batched. However, losses of F are not expected to drastically change the F environments observed.

### 3.2. Simulated spectra

**Figure 5:**
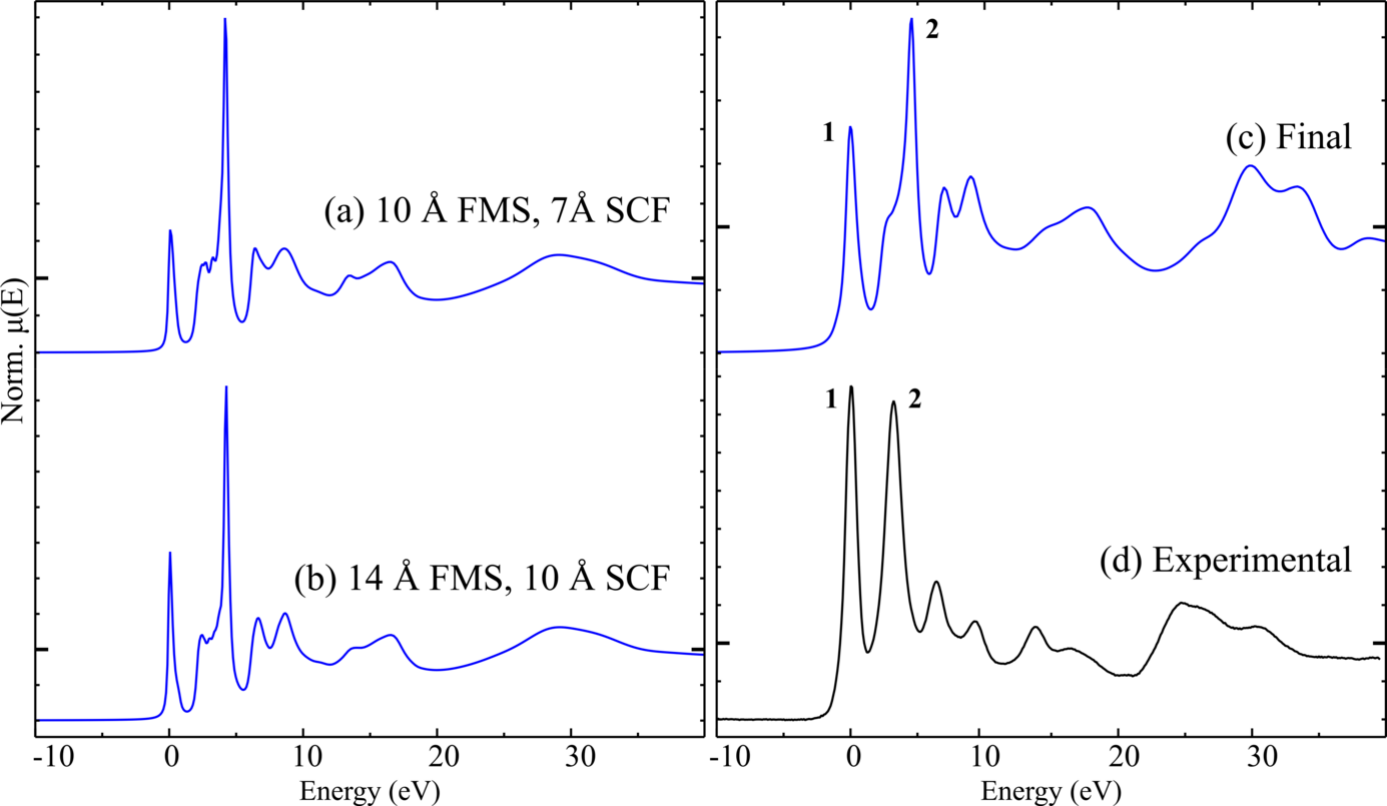
Calculated and experimental F *K*-edge spectra of CaF_2_: (*a*) preliminary calculated spectrum with calculation radii of 10 Å and 7 Å for FMS and SCF, respectively; (*b*) calculated spectrum with calculation radii of 14 Å and 10 Å; (*c*) final calculated spectrum with additional spectral broadening; (*d*) normalized experimental spectrum (TEY). Each spectrum is presented on a unique *y*-axis, with μ(*E*) = 1 marked with a larger tick.

The possibility of calculating F *K*-edge spectra with a user-friendly approach and a minimum of assumptions or prior knowledge was attempted with the *FEFF10* software package, with the accurate reproduction of the spectrum of CaF_2_ used as a test case. Initial simulated spectra calculated with default or standard parameters appeared to partially reproduce the experimental spectrum [see Fig. 5 ▸(*a*)], so a campaign of further simulations was undertaken to understand the computational parameters necessary for simulation of the F *K*-edge in these inorganic systems.

To start, the impact of the self-consistent field (SCF) and full multiple scattering (FMS) radii was tested. Convergence with respect to the SCF radius was reached at a cluster radius of between 9 and 10 Å (10 Å corresponds to 307 atoms, 19 coordination shells), though only slight changes were seen past an SCF radius of 7 Å; this radius was used for less computationally demanding rapid scoping trials. An SCF radius of 10 Å was used for all final calculated spectra.

Convergence with respect to the FMS calculation radius was more complex, with convergence proper not observed in the range tested. Near-convergence, where only negligible changes occur between clusters of differing radii, was reached at an FMS radius between 13 and 14 Å (14 Å corresponds to 863 atoms, 36 coordination shells). As the FMS radius increases, scattering paths involving more distant atoms will contribute to the multiple scattering processes, resulting in a higher number of possibly observable spectral features; calculated spectra reproduced major features as the FMS radius increased to 9 Å (227 atoms, 15 shells) and higher. All features observed experimentally were at least reasonably replicated by the calculated spectrum at an FMS radius of 14 Å, suggesting that distant scatterers are important contributors to the final spectrum, in agreement with previous observations (Hudson *et al.*, 1994 ▸). Further, the lack of substantial core-hole broadening and beamline resolution enables examination of many slight features whose contribution is more difficult to identify on *K*-edge spectra from higher atomic number elements. A comparison between the experimental data and a preliminary calculated spectrum after convergence of the calculation radii only is shown in Fig. 5 ▸(*b*). It should be noted that calculation time increases following the third power of the number of atoms (Ankudinov *et al.*, 1998 ▸), with the FMS calculation with radius 14 Å taking over 13× longer than with radius 10 Å.

During calculations of the Fermi energy, *E*_f_, *FEFF* is known to have uncertainties on the order of 1 eV, so a correction term is added in the term describing the exchange-correlation potential, accounting for the difference between the calculated *E*_f_ and that observable as a gap in the calculated density of states. This correction results in the relative intensities of the two white-line features **1** and **2** (see above, Fig. 1 ▸) better approximating the features seen experimentally (see Fig. S3).

Three different core-hole models are currently possible within *FEFF*, and all were tested, though little significant improvement was seen in the simulated spectra (see Fig. S4). Given that the presence of a core-hole would be expected to have an impact on the spectrum of insulating CaF_2_, it was decided that the final state rule (FSR) description would be used for further simulations.

Different exchange-correlation potential models were tested, though models other than the default implementation for XANES in *FEFF* (Hedin-Lundqvist potential for the fine structure, ground state potential for the atomic background) led to significantly less accurate calculated spectra. The use of a density-dependent many-pole self-energy (MPSE) model, with the loss function also calculated within *FEFF*, did lead to improvements in the reproduction of higher energy features (see Fig. S5), and was utilized in further calculations thereafter.

Finally, a small amount of spectral broadening, in addition to the tabulated core-hole lifetime broadening, further improved reproduction of the experimental spectrum. This was implemented by the additional of an imaginary component of 0.2 eV to the exchange-correlation potential. Given that the reported resolving power of the SGM beamline is greater than 5000 (Δ*E*/*E*) (Regier *et al.*, 2007 ▸), which corresponds to ∼0.14 eV at the F *K*-edge, the 0.2 eV imaginary component appears to be a reasonable estimate of the experimental contribution to the spectral broadening.

The addition of many other functions and corrections was also tested, though they did not improve agreement with the experimentally observed spectrum.

The final calculated spectrum [see Fig. 5 ▸(*c*)] reproduces all features observed in the experimental spectrum, though with some differences in relative intensity; see, for example, the features **1** and **2**. A difference in the overall edge position is also apparent, with feature **1** at 689.4 eV in the experimental spectrum and 684.0 eV in the calculated spectrum (note that spectra in Fig. 5 ▸ are plotted in relative energy from the edge). Similarly, there are slight inaccuracies in the relative positions of post-edge features, with the calculated spectrum appearing ‘stretched’ towards higher energies. Despite these common limitations, it is apparent that the calculated spectrum is in good agreement with the experimental data.

In order to validate the procedure and parameters refined for the F *K*-edge spectrum of CaF_2_, the F *K*-edge spectrum of cuspidine (Ca_4_Si_2_O_7_F_2_) was also simulated. The structure of cuspidine contains two unique F sites, both fourfold coordinated by Ca and of multiplicity four; the spectrum of each is simulated separately, then averaged. The spectrum showed only slight changes as the SCF radius was increased, with convergence resulting at a cluster radius of between 9 and 10 Å (10 Å corresponds to 319 atoms around one F site, and 323 atoms around the other). As above, near-convergence with respect to the FMS radius was complete at a radius of between 12 and 13 Å (13 Å corresponds to 683 atoms around one F site, 679 around the other), though changes past 10 Å were small.

**Figure 6:**
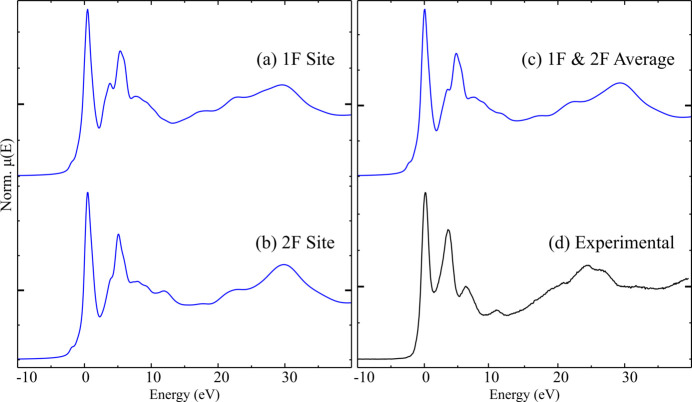
Calculated and experimental F *K*-edge spectra of cuspidine, Ca_4_Si_2_O_7_F_2_: (*a*) calculated spectrum of the 1F site with calculation radii of 13 Å and 10 Å for FMS and SCF, respectively; (*b*) calculated spectrum of the 2F site with calculation radii of 13 Å and 10 Å; (*c*) average of (*a*) and (*b*), the final calculated spectrum; (*d*) normalized experimental spectrum (PFY). Each spectrum is presented on a unique *y*-axis, with μ(*E*) = 1 marked with a larger tick.

The final calculated spectra (FMS radius, 13 Å; SCF radius 10 Å) for the two F sites are similar, with only slight differences in the shape of the second major spectral feature at a relative energy of ∼5 eV and the relative intensity of the high energy feature at a relative energy ∼30 eV [see Figs. 6 ▸(*a*) and 6(*b*)]. Both F sites are of multiplicity four, so the final calculated spectrum is a simple average of the two [Fig. 6 ▸(*c*)] and closely resembles the individual component spectra. As seen for CaF_2_, a difference in energy positions of the experimental (white-line maximum at 689.5 eV) and calculated spectra (white-line maximum at 685.5 eV) was apparent, and the post-edge features are ‘stretched’ to higher energies in the calculated spectrum. The shape of the features forming the post-white-line maximum (relative energy ∼5 eV) also differs, though there is still excellent agreement in the shapes of the overall spectral envelopes.

## 4. Discussion

It should be apparent that the F *K*-edge is rich in information for many of these complex inorganic systems, though there can be difficulties in relating the observed spectral features to the underlying F environments, as in the LPB40 glass discussed above. The small core-hole lifetime broadening of the F *K*-edge is key to many of the observations and analyses shown here.

Fingerprinting of the observed spectra has been shown to be a powerful technique, for both simple systems such as FLiNa and more complex systems such as the glasses SSPN15 and CS-Stebbins. Materials have distinct features relating to the F environment(s) present, with the features and overall shape of the white line permitting identification of the major F coordination by simple visual comparison. Similarly, the application of LCF and related analyses is clearly applicable to many systems, though the same spectral complexity that makes it useful can also lead to difficulties in consistent background subtraction, a significant source of uncertainty in LCF. As is particularly obvious for the spectra of the glassy systems above, the spectral broadening caused by structural disorder is varied, with the spectrum of CS-Stebbins significantly broader than that of either of the crystalline references, whereas that of SSPN15 was extremely similar to the spectrum of Na_2_PO_3_F with no additional broadening apparent.

Some drawbacks of F *K*-edge XAS include the particularly surface sensitive nature of electron yield measurements, including the drain current TEY measurements shown here. All measurements here, even PFY measurements, are only probing the top few micrometres of most materials; the absorption length of 700 eV X-rays in Ca_4_Si_2_O_7_F_2_ is less than 0.3 µm, for example. For non-conducting and/or concentrated samples, ensuring a small particle size sample in good contact with a conductive backing material can be vital to collecting spectra of useful quality. This is particularly important for TEY measurements, as insulating materials, especially with larger particle sizes, can display anomalous regions of negative absorption after the absorption edge if there is a significant build-up of surface charge (Achkar *et al.*, 2011 ▸).

Overall, TEY measurements at the F *K*-edge are generally more successful for high F content, concentrated materials, avoiding the issues with over-absorption (*i.e.* self-absorption) seen in some of the PFY spectra reported here. On the other hand, low F content (dilute) samples often exhibited essentially no TEY signal, especially where high fractions of O (with the O *K*-edge at 543.1 eV) led to high background absorption. In these cases, the PFY measurements were better, with no impact from self-absorption since these had the lower F concentrations.

In some systems, iPFY measurements could be utilized as a probe for highly absorbing materials where the TEY signal is not viable. However, the iPFY spectrum of AlF_3_·3H_2_O (see Fig. 2 ▸) appeared to be somewhat attenuated and more similar to the PFY detected spectrum than the TEY one for the same material. This could be due to the inability to separate the O *K*α emission from the F *K*α emission due to poor detector energy resolution, though the overlap was only slight (see Fig. S2); if this is the root cause, this would likely not be an issue for edges with a larger separation in energy. The signal-to-noise ratio of the O *K*α emission was also relatively poor, which may have led to the poor resolving of spectral features. Alternatively, there may be some material difference in the extreme surface layer probed by TEY compared with the deeper ‘bulk’ probed by PFY and iPFY, though it is unclear what form this may take in the already fully hydrated AlF_3_·3H_2_O. If the apparent spectral distortions are caused by issues with the iPFY detection, it may be remedied by the use of a more complex resonant inelastic X-ray scattering (RIXS)-style emission spectrometer, as has been previously performed successfully (Gotz *et al.*, 2012 ▸), using the O *K*α emission as the iPFY signal for the Fe *L*_2_- and *L*_3_-edges.

As was seen unintentionally for the glass CS-Stebbins, at the soft X-ray energies relevant here, there are many other absorption edges close by, with the Fe *L*_3_- and *L*_2_-edges (706.8 eV and 719.9 eV tabulated energies) seen superimposing the F *K*-edge EXAFS where it is present. This can of course cause issues with analysis of the F *K*-edge, but in some cases it may be possible to collect the spectra of multiple absorption edges in one measurement or in quick succession, permitting simultaneous or rapid evaluation of both, for example, the F environment and Fe oxidation state.

## 5. Conclusions

A series of inorganic compounds, minerals, and glasses were studied using F *K*-edge X-ray absorption spectroscopy. The nature of the small core-hole broadening at this edge leads to many well resolved multiple scattering features in the XANES region, particularly in binary fluorides and other highly crystalline materials. Interpretation of these features in principle can be obtained by electronic calculations such as those implemented in the *FEFF* software suite. For best results, materials studied should have low Fe content due to the nearby Fe *L*_2,3_-edges, and powders should have small particles in good contact with a conductive sample holder. The alkali and alkaline-earth binary fluorides show the expected shifts in edge position due to increasing ionicity when going to heavier cations, as well as differences in spectral shape as a result of differences in both structure and orbital hybridization. Pseudo-binary (Na/Ca)–Al–fluorides show a richness of spectral shape depending on the exact details of the different F local geometries and coordination, and their relative abundance within the structure. F-containing glasses sometimes resemble their constituent fluoride spectra, but in other cases are significantly different, due to variation in the F environments seen in the specific glass and fluoride. This study forms a solid basis for the focused collection of high-quality F *K*-edge spectra on a much broader range of material systems than have yet to be investigated with this technique, with a particular focus on application towards characterizing complex inorganic materials.

## 6. Related literature

The following references, not cited in the main body of the paper, have been cited in the supporting information: Ravel & Newville (2005 ▸); Toby & Von Dreele (2013 ▸). 

## Supplementary Material

Sections S1 and S2 and Figs S1 to S4 of the supporting information. DOI: 10.1107/S1600577526002821/vy5049sup1.pdf

## Data Availability

Data are available from the corresponding author by reasonable request.

## References

[bb1] Achkar, A. J., Regier, T. Z., Monkman, E. J., Shen, K. M. & Hawthorn, D. G. (2011). *Sci. Rep.* **1**, 182. 10.1038/srep00182 PMC3240986 22355697

[bb3] Ankudinov, A. L., Ravel, B., Rehr, J. J. & Conradson, S. D. (1998). *Phys. Rev. B* **58**, 7565–7576.

[bb4] Annunziato, A., Anelli, F., Du Teilleul, P. L. P., Cozic, S., Poulain, S. & Prudenzano, F. (2022). *Opt. Express* **30**, 44160–44174. 10.1364/OE.471090 36523097

[bb5] Bansal, K., Mishra, N. K., Abdullahi, I., Singh, P. J., Tyagi, M. & Singh, S. (2024). *Opt. Mater.* **147**, 114579.

[bb6] Bessada, C. & Anghel, E. M. (2003). *Inorg. Chem.* **42**, 3884–3890. 10.1021/ic026074o 12793826

[bb7] Body, M., Silly, G., Legein, C., Buzaré, J.-Y., Calvayrac, F. & Blaha, P. (2005). *J. Solid State Chem.* **178**, 3655–3661.

[bb8] Brow, R. K., Osborne, Z. A. & Kirkpatrick, R. J. (1992). *J. Mater. Res.* **7**, 1892–1899.

[bb9] Burke, F. M., Ray, N. J. & McConnell, R. J. (2006). *Int. Dent. J.* **56**, 33–43. 10.1111/j.1875-595x.2006.tb00072.x 16515011

[bb10] Cattaneo, A. S., Lima, R. P., Tambelli, C. E., Magon, C. J., Mastelaro, V. R., Garcia, A., de Souza, J. E., de Camargo, A. S. S., de Araujo, C. C., Schneider, J. F., Donoso, J. P. & Eckert, H. (2008). *J. Phys. Chem. C* **112**, 10462–10471.

[bb11] Chen, C., Jiang, Y., Zhang, L., Guan, F., Wang, Z., Huang, X., Zeng, H., Yuan, X., Zhang, L. & He, J. (2022). *J. Am. Ceram. Soc.* **105**, 2595–2604.

[bb12] Christie, J. K., Pedone, A., Menziani, M. C. & Tilocca, A. (2011). *J. Phys. Chem. B* **115**, 2038–2045. 10.1021/jp110788h 21322627

[bb13] Daniel, P., Bulou, A., Rousseau, M., Nouet, J., Fourquet, J. L., Leblanc, M. & Burriel, R. (1990). *J. Phys. Condens. Matter* **2**, 5663–5677. 10.1103/physrevb.42.10545 9995314

[bb14] Domesle, R. & Hoppe, R. (1980). *Z. Krist.* **153**, 317–328.

[bb15] Durand, J., Cot, L. & Galigné, J. L. (1974). *Acta Cryst.* B**30**, 1565–1569.

[bb16] Galdo, E., Cozic, S., Szymczyk, L., Chevire, F., Lebullenger, R., Gautier, A., Calvez, L., Poulain, S., Poulain, M. & Troles, J. (2025). *Opt. Mater. Expr.* **15**, 1189–1195.

[bb17] Gandy, D. (2019). *Program on Technology Innovation: Material Property Assessment and Data Gap Analysis for the Prospective Materials for Molten Salt Reactors – Research and Development.* Palo Alto, CA: EPRI.

[bb18] Gharbi, A., Oudadesse, H., Ashammakhi, N., Cheikhrouhou-Koubaa, W., Blaeser, A., Rau, J. V., Antoniac, I., Derbel, N. & El Feki, H. (2023). *Ceram. Int.* **49**, 18238–18247. 10.3390/jfb14070364 PMC10381889 37504859

[bb19] Gotz, M. D., Soldatov, M. A., Lange, K. M., Engel, N., Golnak, R., Könnecke, R., Atak, K., Eberhardt, W. & Aziz, E. F. (2012). *J. Phys. Chem. Lett.* **3**, 1619–1623. 10.1021/jz301665s 26291098

[bb20] Guo, J., Li, L., Chen, J., Li, H. & Guo, H. (2024). *J. Alloys Compd.* **980**, 173670.

[bb21] Hawthorne, F. C. & Herwig, S. (2021). *Can. Mineral.* **59**, 211–241.

[bb22] Hill, R. G., Stamboulis, A. & Law, R. V. (2006). *J. Dent.* **34**, 525–532. 10.1016/j.jdent.2005.08.005 16522349

[bb23] Hudson, E., Moler, E., Zheng, Y., Kellar, S., Heimann, P., Hussain, Z. & Shirley, D. A. (1994). *Phys. Rev. B* **49**, 3701–3708. 10.1103/physrevb.49.3701 10011259

[bb24] Huve, L., Delmotte, L., Martin, P., Le Dred, R., Baron, J. & Saehr, D. (1992). *Clays Clay Miner.* **40**, 186–191.

[bb25] Jacoboni, C., Leble, A. & Rousseau, J. J. (1981). *J. Solid State Chem.* **36**, 297–304.

[bb26] Jacobsen, M. J., Balić -Žunić, T., Mitolo, D., Katerinopoulou, A., Garavelli, A. & Jakobsson, S. P. (2014). *Miner. Mag.* **78**, 215–222.

[bb27] Kas, J. J., Vila, F. D., Pemmaraju, C. D., Tan, T. S. & Rehr, J. J. (2021). *J. Synchrotron Rad.* **28**, 1801–1810. 10.1107/S1600577521008614 34738933

[bb28] Kemnitz, E., Groß, U., Rüdiger, S., Scholz, G., Heidemann, D., Troyanov, S. I., Morosov, I. V. & Lemée-Cailleau, M. (2006). *Solid State Sci.* **8**, 1443–1452.

[bb29] Keski-Rahkonen, O. & Krause, M. O. (1974). *At. Data Nucl. Data Tables* **14**, 139–146.

[bb30] Kiczenski, T. J. & Stebbins, J. F. (2002). *J. Non-Cryst. Solids* **306**, 160–168.

[bb31] Labouriau, A., Kim, Y.-W., Chipera, S., Bish, D. L. & Earl, W. L. (1995). *Clays Clay Miner.* **43**, 697–704.

[bb32] Liu, J., Zhao, X., Xu, Y., Wu, H., Xu, X., Lu, P., Zhang, X., Zhao, X., Xia, M., Tang, J. & Niu, G. (2023). *Laser Photon. Rev.* **17**, 2300006.

[bb33] Lumley, R. (2010). *Fundamentals of Aluminium Metallurgy: Production, Processing and Applications.* Cambridge: Elsevier Science & Technology.

[bb34] Martel, L., Capelli, E., Body, M., Klipfel, M., Beneš, O., Maksoud, L., Raison, P. E., Suard, E., Visscher, L., Bessada, C., Legein, C., Charpentier, T. & Kovács, A. (2018). *Inorg. Chem.* **57**, 15350–15360. 10.1021/acs.inorgchem.8b02683 30475605

[bb35] McCloy, J. S., Bussey, J. M. & Dixon Wilkins, M. C. (2025). *MRS Adv.* **10**, 1868–1873.

[bb36] McCloy, J. S., Smith-Gray, N., Bussey, J. M., Stone-Weiss, N. & Youngman, R. E. (2024). *Inorg. Chem.* **63**, 4669–4680. 10.1021/acs.inorgchem.3c04281 38394614

[bb37] Nakai, S., Ohashi, M., Mitsuishi, T., Maezawa, H., Oizumi, H. & Fujikawa, T. (1986). *J. Phys. Soc. Jpn* **55**, 2436–2442.

[bb38] Newville, M. (2013). *J. Phys. Conf. Ser.* **430**, 012007.

[bb2] Nowroozi, M. A., Mohammad, I., Molaiyan, P., Wissel, K., Munnangi, A. R. & Clemens, O. (2021). *J. Mater. Chem. A* **9**, 5980–6012.

[bb39] Oizumi, H., Fujikawa, T., Ohashi, M., Maezawa, H. & Nakai, S. (1985). *J. Phys. Soc. Jpn* **54**, 4027–4033.

[bb40] Olmi, F., Sabelli, C. & Trosti-Ferroni, R. (1993). *Eur. J. Mineral.* **5**, 1167–1174.

[bb41] Patro, L. N. & Hariharan, K. (2013). *Solid State Ionics* **239**, 41–49.

[bb42] Poulain, M. (2024). *APL Photon.* **9**, 091103.

[bb43] Ravel, B. & Newville, M. (2005). *J. Synchrotron Rad.* **12**, 537–541. 10.1107/S0909049505012719 15968136

[bb44] Reben, M. & Środa, M. (2013). *J. Therm. Anal. Calorim.* **113**, 77–81.

[bb45] Regier, T., Krochak, J., Sham, T. K., Hu, Y. F., Thompson, J. & Blyth, R. I. R. (2007). *Nucl. Instrum. Methods Phys. Res. A* **582**, 93–95.

[bb46] Roesch, P., Vogel, C., Huthwelker, T., Wittwer, P. & Simon, F.-G. (2022). *Environ. Sci. Pollut. Res.* **29**, 26889–26899. 10.1007/s11356-021-17838-z PMC8989862 34860340

[bb47] Roesch, P., Vogel, C., Wittwer, P., Huthwelker, T. N., Borca, C., Sommerfeld, T., Kluge, S., Piechotta, C., Kalbe, U. & Simon, F.-G. (2023). *Environ. Sci. Process. Impacts* **25**, 1213–1223. 10.1039/d3em00107e 37335293

[bb48] Roper, R., Harkema, M., Sabharwall, P., Riddle, C., Chisholm, B., Day, B. & Marotta, P. (2022). *Ann. Nucl. Energy* **169**, 108924.

[bb49] Sadoc, A., Body, M., Legein, C., Biswal, M., Fayon, F., Rocquefelte, X. & Boucher, F. (2011). *Phys. Chem. Chem. Phys.* **13**, 18539–18550. 10.1039/c1cp21253b 21947333

[bb50] Sangster, J. & Pelton, A. D. (1987). *J. Phys. Chem. Ref. Data* **16**, 509–561.

[bb51] Sanz-Matias, A., Roychoudhury, S., Feng, X., Yang, F., Kao, L. C., Zavadil, K. R., Guo, J. & Prendergast, D. (2022). *Chem. Mater.* **34**, 9144–9158.

[bb52] Schaller, T., Dingwell, D. B., Keppler, H., Knöller, W., Merwin, L. & Sebald, A. (1992). *Geochim. Cosmochim. Acta* **56**, 701–707.

[bb53] Schroeder, S. L. & Weiher, N. (2006). *Phys. Chem. Chem. Phys.* **8**, 1807–1811. 10.1039/b518124k 16633665

[bb54] Shah, F. A. (2016). *Mater. Sci. Eng. C* **58**, 1279–1289. 10.1016/j.msec.2015.08.064 26478431

[bb55] Stamboulis, A., Hill, R. G. & Law, R. V. (2005). *J. Non-Cryst. Solids* **351**, 3289–3295.

[bb56] Stebbins, J. F. & Zeng, Q. (2000). *J. Non-Cryst. Solids* **262**, 1–5.

[bb57] Sun, Z., Huang, X., Yang, J., Wang, S. & Wu, S. (2023). *Ceram. Int.* **49**, 15500–15506.

[bb58] Toby, B. H. & Von Dreele, R. B. (2013). *J. Appl. Cryst.* **46**, 544–549.

[bb59] Tossell, J. A. & Liu, Y. (2004). *Magn. Reson. Chem.* **42**, S34–S40. 10.1002/mrc.1434 15366039

[bb60] Tröger, L., Arvanitis, D., Baberschke, K., Michaelis, H., Grimm, U. & Zschech, E. (1992). *Phys. Rev. B* **46**, 3283–3289. 10.1103/physrevb.46.3283 10004043

[bb61] Vinogradov, A. S., Fedoseenko, S. I., Krasnikov, S. A., Preobrajenski, A. B., Sivkov, V. N., Vyalikh, D. V., Molodtsov, S. L., Adamchuk, V. K., Laubschat, C. & Kaindl, G. (2005). *Phys. Rev. B* **71**, 045127.

[bb62] Vogel, C., Roesch, P., Wittwer, P., Piechotta, C., Lisec, J., Sommerfeld, T., Kluge, S., Herzel, H., Huthwelker, T., Borca, C. & Simon, F. (2023). *Environ. Sci.: Adv.* **2**, 1436–1445. 10.1039/d3em00107e 37335293

[bb63] Ward, J. D., Bowden, M., Tom Resch, C., Eiden, G. C., Pemmaraju, C. D., Prendergast, D. & Duffin, A. M. (2017). *At. Spectrosc.* **127**, 20–27.

[bb64] Wiegand, A., Buchalla, W. & Attin, T. (2007). *Dent. Mater.* **23**, 343–362. 10.1016/j.dental.2006.01.022 16616773

[bb65] Yan, B., Wang, J. & Liu, J. (2021). *Water Res.* **201**, 117371. 10.1016/j.watres.2021.117371 34186289

[bb66] Yang, H., Ghose, S. & Hatch, D. M. (1993). *Phys. Chem. Miner.* **19**, 528–544.

[bb67] Youngman, R. (2018). *Materials* **11**, 476. 10.3390/ma11040476 PMC5951322 29565328

[bb68] Zhou, B., Sherriff, B. L., Hartman, J. S. & Wu, G. (2007). *Am. Mineral.* **92**, 34–43.

