# Supplementary material for: F K-edge XAS as a tool to examine F environments in complex inorganic systems
Source: J Synchrotron Radiat. 2026 Apr 10;33(Pt 3):715–24. doi: 10.1107/S1600577526002821 (PMC13148593; doi:10.1107/S1600577526002821)
Supplement: Supplementary file 1 [file s-33-00715-sup1.pdf]

JOURNAL OF SYNCHROTRON RADIATION

Volume 33 (2026)

Supporting information for article:

F K-edge XAS as a tool to examine F environments in complex inorganic systems

Malin C. Dixon Wilkins, John M. Bussey and John S. McCloy

S1. Additional Methodology

The amorphous nature of the glasses and phase purity of the produced crystalline phases was established by powder X-ray diffraction, utilising a Panalytical X'pert Pro diffractometer with Ni-filtered Cu Kα radiation. Rietveld-method refinements in the GSAS-II software suite (Toby & Von Dreele, 2013) were used to estimate the phase purity of the crystalline materials.

$CaAlF_5$ and Ca_2AlF_7 (synthetic sbacchiite) were synthesized from CaF_2 and nominally anhydrous AlF_3. Stoichiometric amounts of the constituent fluorides were mixed by hand grinding in a mortar and pestle, then pressed into a pellet. The pellet was placed into a Ni foil capsule that was manually sealed by repeatedly folding the open end of the capsule onto itself. The sealed capsule was buried in a bed of $CaCO_3$ to neutralize any HF gas produced. For $CaAlF_5$, the assembly was placed into a furnace at 750 °C for 1 h before being removed from the furnace to cool to room temperature. For Ca_2AlF_7, the assembly was placed into a furnace at 750 °C, the temperature increased to 850 °C over the course of 15 min, and after 3 min at 850 °C the materials were removed from the furnace to cool to room temperature.

The major phase in $CaAlF_5$ was β-$CaAlF_5$ (~80 wt.%) (Body *et al.*, 2005), alongside lesser amounts of Ca_2AlF_7 (~14 wt.%), CaF_2 (~1 wt.%) and Al_2O_3 (~5 wt.%). The major phase in Ca_2AlF_7 was Ca_2AlF_7 (~89 wt.%) (Domesle & Hoppe, 1980), alongside lesser amounts of CaF_2 (~5 wt.%), $CaAlF_5$ (~3 wt.%), Al_2O_3 (~3 wt.%), and $CaCO_3$ (<1 wt.%).

$Ca_4Si_2O_7F_2$ (synthetic cuspidine) was synthesize from CaF_2, $CaCO_3$ and SiO_2. Stoichiometric amounts of the constituent fluorides were mixed by hand grinding in a mortar and pestle. The resulting powder was heat treated in air in a Pt10Rh crucible at 1300 °C for 1 h.

The glass SSPN15 (mol.%, 10SnO-40SnF_2-35P_2O_5-15NaF) (Chen *et al.*, 2022) was prepared from SnO, SnF_2, P_2O_5 and NaF by melting of stoichiometric amounts of the precursors in a covered alumina crucible. The glass was melted once at 450 °C for 0.5 h before being quenched by pouring onto an Inconel plate.

The glass CS-Stebbins (mol.%, 39.2CaO-58.9SiO_2-2.0CaF_2) (Stebbins & Zeng, 2000) was prepared from $CaCO_3$, SiO_2 and CaF_2 by melting of stoichiometric amounts of the precursors in a covered Pt10Rh crucible. The glass was melted once at 1650 °C for 1 h before being quenched by dipping the bottom of the crucible in room temperature water. It should be noted that the SiO2 used (US Silica, Lot# 08062110) has a known Fe contamination on the order of 10s of ppm Fe2O3.

The glass LPB40 (mol.%, 50B_2O_3-10PbO-40LiF) (Cattaneo *et al.*, 2008) was prepared from B_2O_3, PbO and LiF by melting of stoichiometric amounts of the precursors in a covered silica crucible. The glass was melted once at 900 °C for 1 h before being quenched by pouring onto an Inconel plate.

All glasses produced were X-ray amorphous.

Suppliers of the synthetic fluorides studied were:

LiF – 99.99%, Prochem

NaF – 99.99%, Alfa Aesar

KF – 99%, Acros Organics

RbF – 99.1%, Thermo Scientific

CsF – 99%, Thermo Scientific

MgF_2 – Technical Grade, Sigma-Aldrich

CaF_2 – Certified (Lot 134438), Fisher Scientific

SrF_2 – 99.9%, Sigma Aldrich

BaF_2 – 99%, Thermo Scientific

SnF_2 – 97.5%, Alfa Aesar

Na_2PO_3F – 95%, AK Scientific

KBF_4 – 99%, Thermo Fisher

AlF_3 (anhydrous) – 99.99%, Prochem

$AlF_3 \cdot 3H_2O$ – 99.99%, Alpha Aesar

The phases present in these materials were checked by powder XRD.

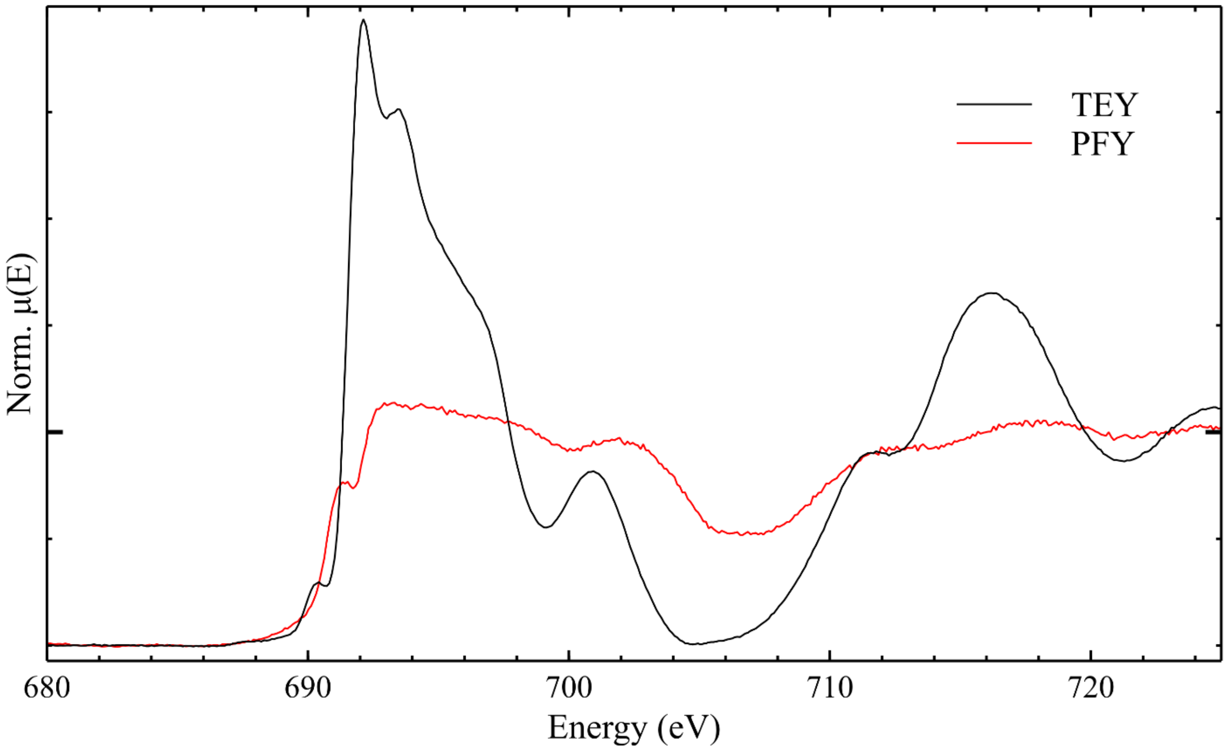

Figure S1 Normalized F K-edge spectra of LiF, collected in both total electron yield (TEY, black) and partial fluorescence yield (PFY, red), showing significant over-absorption (also known as self-absorption) in the PFY signal.

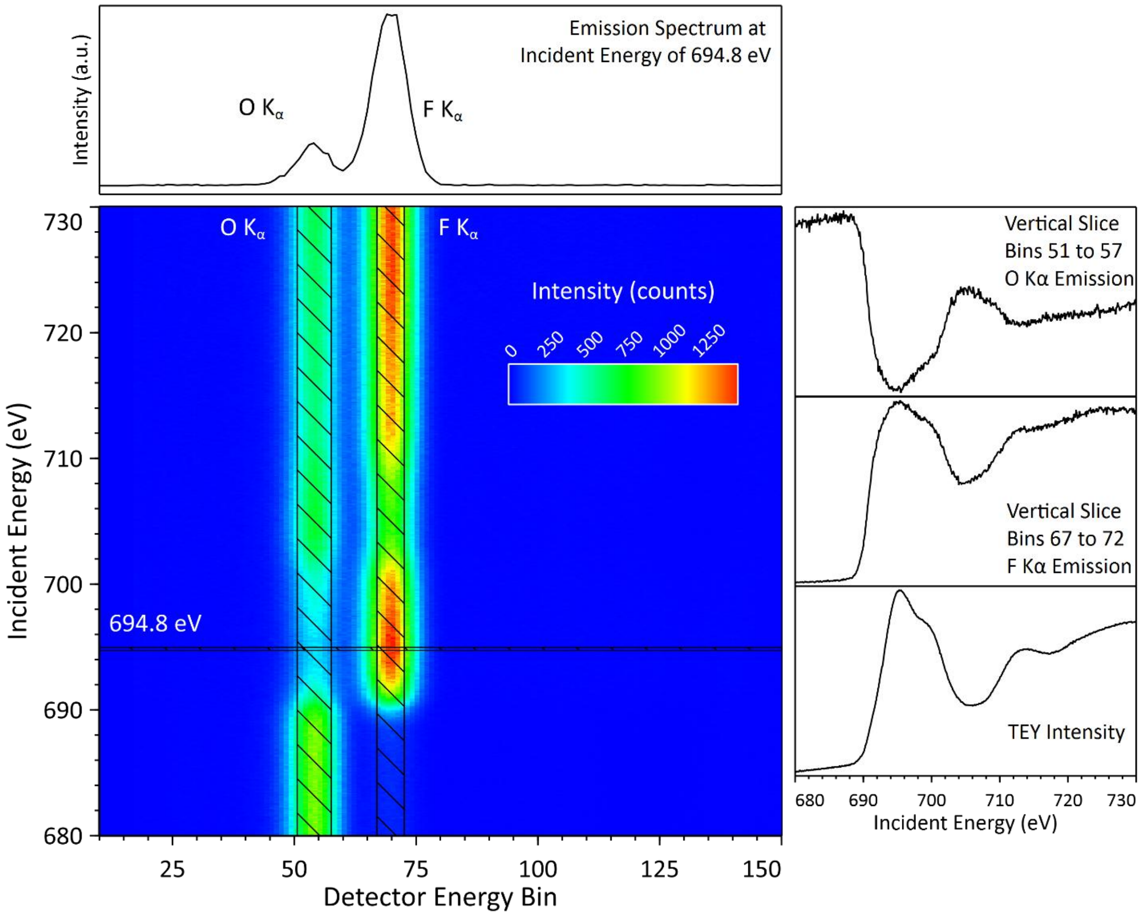

Figure S2 Contour plot of the response of a single detector as the incident energy was scanned over the F K-edge of a sample of $AlF_3{\cdot}3H_2O$. (Right) Two vertical slices showing integration over the detector energy bins corresponding to the O Kα and F Kα emissions, as well as the TEY intensity monitored over the same scan in energy. (Top) Horizontal slice corresponding to an incident energy of 694.8 eV, showing the presence of both O and F Kα emissions.

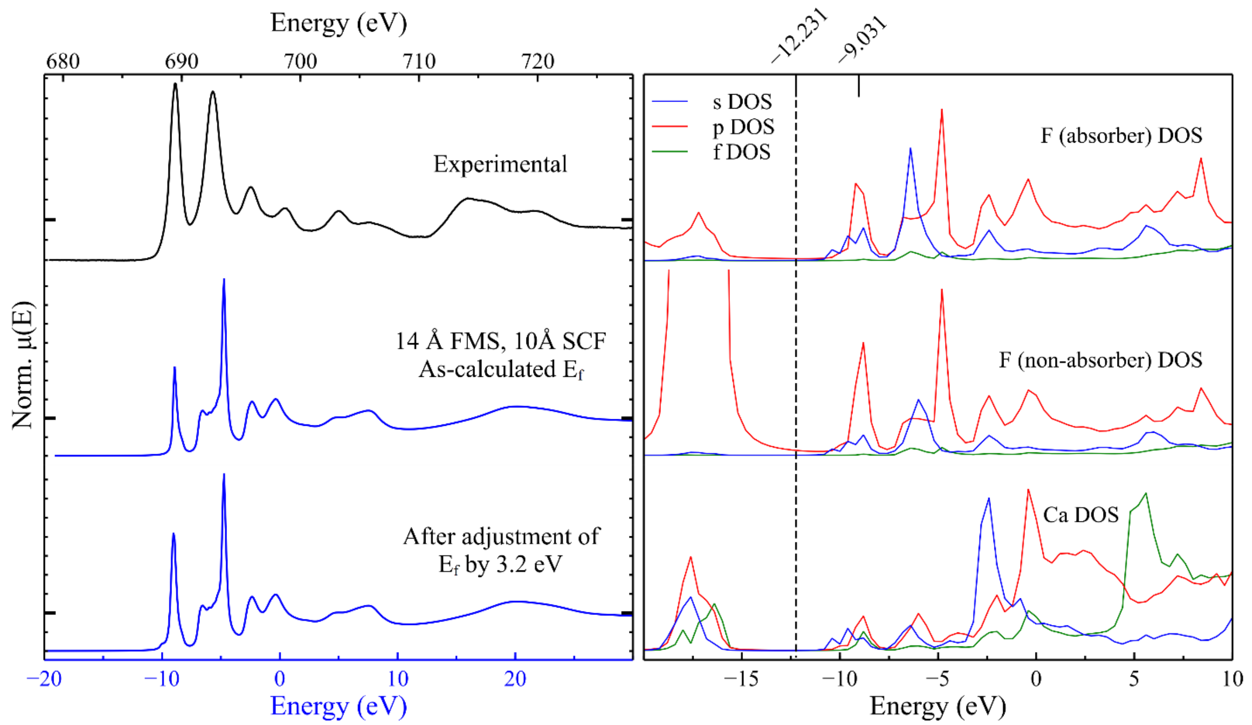

Figure S3 (Left) Normalised experimental (TEY) and as-calculated F K-edge spectra of CaF_2, with and without correction of E_f applied during calculation. (Right) Calculated *s*, *p* and *d* density of states (DOS) for Ca and both absorber and non-absorber F. Each spectrum is presented on a unique y-axis, with μ(E) = 1 marked with a larger tick.

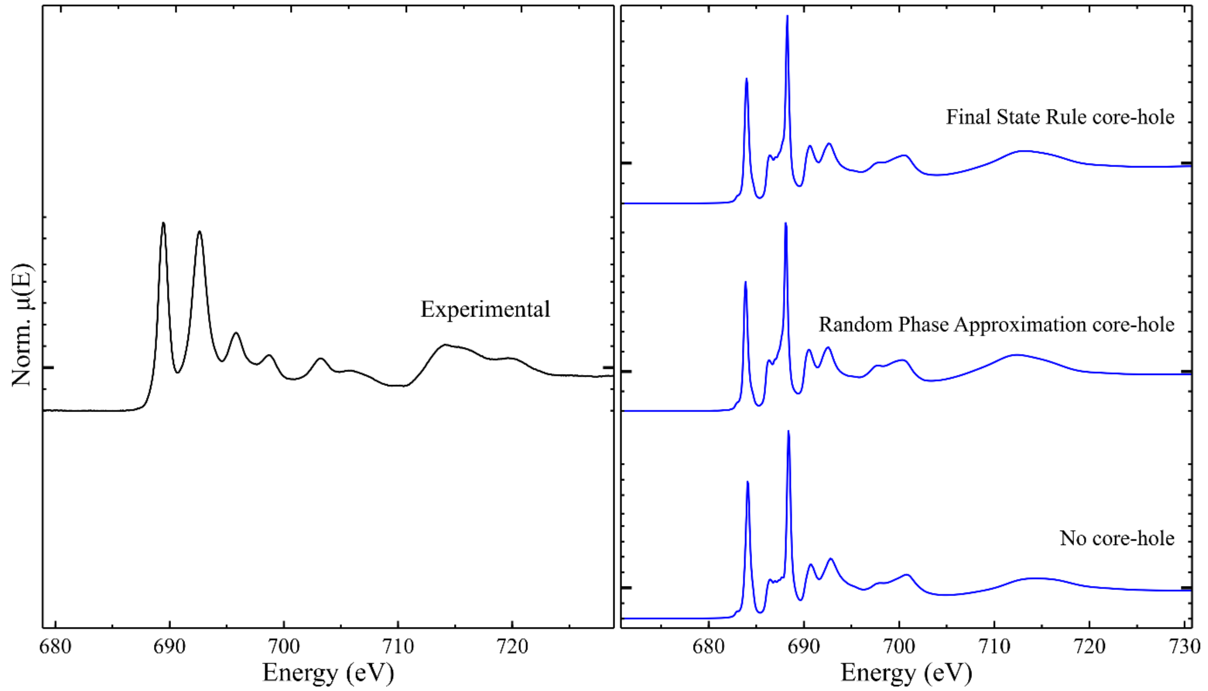

Figure S4 (Left) Normalised experimental TEY and (right) calculated F K-edge spectra of CaF_2. Calculated spectra are labelled with the core-hole model utilized. Each spectrum is presented on a unique y-axis, with μ(E) = 1 marked with a larger tick.

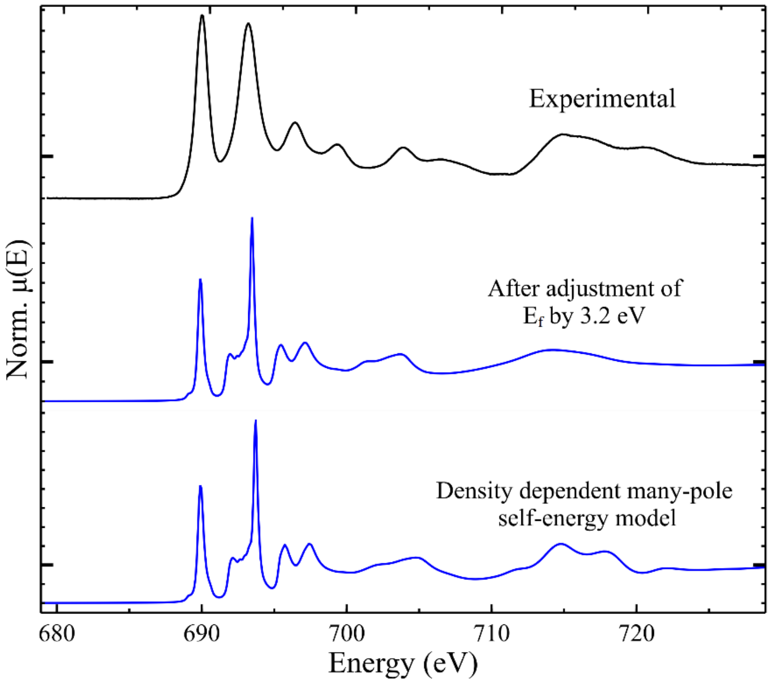

Figure S5 (Upper) Normalised experimental TEY and as-calculated (middle, lower) F K-edge spectra of CaF_2. Spectra were calculated without (middle) and with (lower) a density dependent many-pole self-energy model. Each spectrum is presented on a unique y-axis, with μ(E) = 1 marked with a larger tick.

S2. Example FEFF input files for CaF_2 and $Ca_4Si_2O_7F_2$

The input files below resulted in the final spectra for CaF_2 and $Ca_4Si_2O_7F_2$, including the different parameters and contributions discussed in the main text, see Figure 5 and Figure 6. The lists of atoms are truncated for clarity as they contain over 2000 atoms; these lists were generated from the structures of CaF_2 and $Ca_4Si_2O_7F_2$ by the Stand-alone Atoms program, part of the Demeter software suite (Ravel & Newville, 2005), the cluster size was set to 20 Å to ensure there were a significant number of atoms available in excess of the FMS and SCF radii.

S2.1. FEFF Input File for simulating F K-edge spectrum of CaF_2

```
TITLE  CaF2
EDGE K
CONTROL 1 1 1 1 1 1
PRINT 1 1 1 1 1 1

LDOS -20 20 0.1
FMS 14 0 2
SCF 10 0 100 0.2 1
S02 0.0
RPATH -1
EXCHANGE 0 3.2 0.2 2
```

```
COREHOLE FSR
OPCONS
MPSE 2 100

XANES 5 0.05 0.1

EGRID
e_grid  -10  10  0.1
e_grid last 50 0.2

 POTENTIALS
  * ipot   Z      tag
     0     9      F
     1     9      F
     2     20     Ca

 ATOMS                  * this list contains 2467 atoms
 *    x          y          z     ipot tag          distance
    0.00000    0.00000    0.00000  0  F1           0.00000
   -1.36775    1.36775    1.36775  2  CA1.1        2.36901
    1.36775   -1.36775    1.36775  2  CA1.1        2.36901
    1.36775    1.36775   -1.36775  2  CA1.1        2.36901
   -1.36775   -1.36775   -1.36775  2  CA1.1        2.36901
    2.73550    0.00000    0.00000  1  F1.1         2.73550
   -2.73550    0.00000    0.00000  1  F1.1         2.73550
    0.00000    2.73550    0.00000  1  F1.1         2.73550
    0.00000   -2.73550    0.00000  1  F1.1         2.73550
    0.00000    0.00000    2.73550  1  F1.1         2.73550
    0.00000    0.00000   -2.73550  1  F1.1         2.73550
…          …          …          …          …          …          …          …
    0.00000   -5.47100  -19.14850  1  F1.46       19.91474

 END
```

S2.2. FEFF Input File for simulating F K-edge spectrum of the 1F site of $Ca_4Si_2O_7F_2$

```
TITLE  F1_Cuspidine
EDGE K
CONTROL 1 1 1 1 1 1
PRINT 1 1 1 1 1 1

LDOS -20 20 0.1
FMS 13 0 2
SCF 10 0 100 0.2 1
S02 0.0
RPATH -1
EXCHANGE 0 2.33 0.2 2
COREHOLE FSR

OPCONS
MPSE 2 100

XANES 5 0.05 0.1
```

```
EGRID
e_grid  -10  10  0.1
e_grid last 50 0.2

 POTENTIALS
  * ipot   Z      tag
    0     9      F
    1     9      F
    2     9      F
    3     20     Ca
    4     20     Ca
    5     20     Ca
    6     20     Ca
    7     14     Si
    8     14     Si
    9     8      O
    10     8      O
    11     8      O
    12     8      O
    13     8      O
    14     8      O
    15     8      O

 ATOMS                  * this list contains 2473 atoms
 *    x          y          z     ipot tag          distance
   0.00000    0.00000    0.00000  0  F7           0.00000
  -1.82000    1.42385    0.35031  5  Ca3.1         2.33719
   1.68325    1.40299   -0.81433  4  Ca2.1         2.33770
   0.73710   -0.89571    2.05182  6  Ca4.1         2.35703
  -0.63015   -1.39962   -1.81272  4  Ca2.2         2.37529
   1.05310    0.00337   -2.62705  1  F7.1          2.83027
  -2.50217   -0.03730   -1.33920  2  F8.1          2.83825
  -0.10999    2.79565   -0.98660  13  O13.1        2.96667
  -1.01265    0.48842    2.82986  15  O15.1        3.04502
   2.66751    0.46313    1.62707  14  O14.1        3.15871
…        …          …          …          …          …          …          …
  -1.94191  -12.54219  -15.45646  8  Si6.163      19.99950

 END
```

S2.3. FEFF Input File for simulating F K-edge spectrum of the 2F site of $Ca_4Si_2O_7F_2$

```
TITLE  T2_Cuspidine
EDGE K
CONTROL 1 1 1 1 1 1
PRINT 1 1 1 1 1 1

LDOS -20 20 0.1
FMS 13 0 2
SCF 10 0 100 0.2 1
S02 0.0
RPATH -1
```

```
EXCHANGE 0 2.33 0.2 2
COREHOLE FSR
OPCONS
MPSE 2 100

XANES 5 0.05 0.1

EGRID
e_grid  -10  10  0.1
e_grid last 50 0.2

 POTENTIALS
  * ipot   Z      tag
     0     9      F
     1     9      F
     2     9      F
     3     20     Ca
     4     20     Ca
     5     20     Ca
     6     20     Ca
     7     14     Si
     8     14     Si
     9     8      O
     10     8      O
     11     8      O
     12     8      O
     13     8      O
     14     8      O
     15     8      O


 ATOMS                  * this list contains 2461 atoms
 *    x          y          z     ipot tag          distance
    0.00000    0.00000    0.00000  0  F8            0.00000
    0.73312   -1.04247    1.92456  3  Ca1.1         2.30828
   -0.68217   -1.46114   -1.68951  5  Ca3.1         2.33554
   -1.87201    1.36232    0.47352  4  Ca2.1         2.36317
    1.72107    1.38318   -0.88931  5  Ca3.2         2.38037
    1.03890   -0.07796   -2.57882  2  F8.1          2.78131
   -2.50217   -0.03730   -1.33920  1  F7.1          2.83825
   -0.46694    2.77395   -0.69089  11  O11.1        2.89658
    0.06883   -2.93935    0.19379  10  O10.1        2.94654
    2.52842    0.44775    1.59024  15  O15.1        3.02031
…         …          …          …          …          …          …          …
  12.70315   -4.81991  -14.67621  15  O15.164      19.99982

 END
```
